# A New Statistical Approach for fNIRS Hyperscanning to Predict Brain Activity of Preschoolers’ Using Teacher’s

**DOI:** 10.3389/fnhum.2021.622146

**Published:** 2021-05-07

**Authors:** Candida Barreto, Guilherme de Albuquerque Bruneri, Guilherme Brockington, Hasan Ayaz, Joao Ricardo Sato

**Affiliations:** ^1^Center of Mathematics, Computing and Cognition, Federal University of ABC, Sao Bernardo do Campo, Brazil; ^2^Physics Institute, Federal University of São Paulo, São Paulo, Brazil; ^3^Center for Natural and Human Sciences, Universidade Federal do ABC, Santo André, Brazil; ^4^School of Biomedical Engineering, Science and Health Systems, Drexel University, Philadelphia, PA, United States; ^5^Department of Psychology, College of Arts and Sciences, Drexel University, Philadelphia, PA, United States; ^6^Drexel Solutions Institute, Drexel University, Philadelphia, PA, United States; ^7^Department of Family and Community Health, University of Pennsylvania, Philadelphia, PA, United States; ^8^Center for Injury Research and Prevention, Children’s Hospital of Philadelphia, Philadelphia, PA, United States; ^9^Interdisciplinary Unit for Applied Neuroscience, Federal University of ABC, Sao Bernardo do Campo, Brazil

**Keywords:** hyperscanning, fNIRS, teacher-student interaction, support vector regression, machine learning

## Abstract

Hyperscanning studies using functional Near-Infrared Spectroscopy (fNIRS) have been performed to understand the neural mechanisms underlying human-human interactions. In this study, we propose a novel methodological approach that is developed for fNIRS multi-brain analysis. Our method uses support vector regression (SVR) to predict one brain activity time series using another as the predictor. We applied the proposed methodology to explore the teacher-student interaction, which plays a critical role in the formal learning process. In an illustrative application, we collected fNIRS data of the teacher and preschoolers’ dyads performing an interaction task. The teacher explained to the child how to add two numbers in the context of a game. The Prefrontal cortex and temporal-parietal junction of both teacher and student were recorded. A multivariate regression model was built for each channel in each dyad, with the student’s signal as the response variable and the teacher’s ones as the predictors. We compared the predictions of SVR with the conventional ordinary least square (OLS) predictor. The results predicted by the SVR model were statistically significantly correlated with the actual test data at least one channel-pair for all dyads. Overall, 29/90 channel-pairs across the five dyads (18 channels 5 dyads = 90 channel-pairs) presented significant signal predictions withthe SVR approach. The conventional OLS resulted in only 4 out of 90 valid predictions. These results demonstrated that the SVR could be used to perform channel-wise predictions across individuals, and the teachers’ cortical activity can be used to predict the student brain hemodynamic response.

## Introduction

Hyperscanning is a neuroimaging acquisition concept that consists of simultaneously measuring the brain activities of two or more individuals while interacting to assess the interpersonal neural synchrony (INS) (Mukamel et al., 2005; Hasson et al., 2012; Wang et al., 2018; Balconi et al., 2019). Functional near-infrared spectroscopy (fNIRS) has gained attention in this field, as it is a modern neuroimaging technique with advantages for naturalistic experiments (Balardin et al., 2017; Curtin and Ayaz, 2018). It is less susceptible to movement artifacts than electroencephalography (EEG) and functional magnetic resonance image (fMRI). It allows the investigation of individuals’ brains in less constrained movement conditions, such as daily life tasks (Ayaz et al., 2013; Pinti et al., 2018a,b; Barreto et al., 2020). These advantages make fNIRS an attractive neuroimaging modality to investigate the brain and explore some populations, such as children, who usually present more movement and require fewer constraints during the experiments (McDonald and Perdue, 2018). Hyperscanning studies with fNIRS have brought new insights about the adult-child brain synchronization that could not be explored before due to these limitations (Piazza et al., 2020). For instance, studies that demonstrated neural coupling across parent-child in cooperation tasks and research that showed the effects of stress in the parent-child brain synchronization (Reindl et al., 2018; Azhari et al., 2019; Miller et al., 2019). Those studies required an unconstrained environment since the infant/child cannot be entirely quiet to avoid movement artifacts, in the case of EEG, or even they cannot go inside an fMRI device.

Another field that benefited from the synergy of device portability and hyperscanning acquisition to investigate subjects’ neural coupling is Education. For many years, the relationship between teacher and student has been investigated only in behavioral studies (Battro et al., 2013). A lack in the literature needs to be fulfilled about the neural correlates related to such a meaningful interaction (Battro, 2010). Recent studies have focused on this matter (Dikker et al., 2017). The first study investigating the teacher-students neural coupling was based on performing a Socratic dialog task (Holper et al., 2013). The authors found a correlation between the student’s and teacher’s hemodynamic signals only when the transfer of knowledge was successful. Another study investigated the teacher-learner process through an fNIRS hyperscanning of the pre-frontal cortex (PFC) of teachers and students playing a video game (Takeuchi et al., 2017). They showed evidence that the teacher’s left PFC might be involved in integrating the teacher’s teaching process, and the student’s learning state. fNIRS hyperscanning was also applied to record dyads’ brain activity while learning-teaching a new song (Pan et al., 2018). In this case, brain synchronization occurs when learners observe the instructor’s vocal behavior. Zheng et al. (2018) have demonstrated that teacher-student interaction is a complex process supported by the prediction-transmission hypothesis. According to this, the teacher will predict the state of the student(s) understanding theory before starting any teaching strategy (Kline, 2015). Although this is a theoretical hypothesis that has been considered to explain some aspects of the teaching-learning process, Zheng et al. (2018) introduced the possibility of using the hyperscanning approach to investigate the brain mechanisms that may underlie it. Their research demonstrated neural evidence supporting this hypothesis and indicated that the interbrain synchronization between teacher and student might enhance the teaching results (Zheng et al., 2018; Sun et al., 2020).

The methodological framework used to analyze the data from the fNIRS hyperscanning studies usually relies on classical approaches such as correlations, wavelet transform coherence (WTC), and general linear model (GLM) analysis (Scholkmann et al., 2013; Tachtsidis and Scholkmann, 2016). Typically, those methodologies are applied to investigate the interbrain synchronization (IBS) between the neural signals of the dyads executing cooperation or competition tasks such as the one performed by Cui and Reiss (2012) and Babiloni and Astolfi (2012). For example, two out of the five studies of brain synchronization applied correlations analysis between the oxyhemoglobin (HBO_2_) time series of teachers and students (Holper et al., 2013; Takeuchi et al., 2017). The other three applied the WTC to the hemodynamic measurements to estimate the IBS of teachers and students (Pan et al., 2018; Zheng et al., 2018, 2020). However, the advance of computational processing power and machine learning techniques has allowed alternative methods to provide a deeper understanding of the neural mechanisms underlying such complex processes.

In this proof-of-concept study, we aim to contribute to the methodological field of hyperscanning data analyses. We attempted to predict the brain of one subject using the other subjects’ brain signals as predictors. We chose the teacher-student interaction to illustrate the usefulness of this methodology according to the prediction-transmission hypothesis. We intended to find hemodynamic correlates that might be related to this hypothesis. We exploit the possibility of predicting a student’s brain hemodynamic response using the teacher’s hemodynamic signals as predictors. We applied two regression models, the support vector regression (SVR) and the ordinary least square (OLS) to the HBO_2_ from the PFC and temporal-parietal junction (TPJ) of teachers and preschoolers realizing a teaching-learning task.

## Methods

### Participants

We collected brain signals from eight healthy pairs of teacher-student. Four adults (two males) age from 21 to 28 years; eight children (four boys) aged between 3 and 5 participated in the experiment. Children were recruited by advertisements in a public school close to the university where the experiment was performed. The teachers were tutors from a Science Museum at the University of São Paulo. Three pairs of subjects were excluded due to difficulties during data acquisition, either due to the inability to follow the experimental task or sensor signal unusable in at least one dyad participant. A local ethics committee approved the research, and all participants (teachers and children’s parents) signed a written consent form.

### Experimental Task

The experimental task aims to emulate the teacher-student interaction as described in Brockington et al. (2018). In this task, the teacher presents the mechanisms to sum two numbers by playing a space-race game with the student. The teacher certified that the child could count from 1 to 12 and then explained how to add two natural numbers using matchsticks. They began the race by throwing two dice of six faces, the player who got the highest sum of the outcomes from the two dice started the game. They continued the race by walking the steps according to the sum of the dice numbers. All dyads performed the same task. It was a continued task without a resting period and lasted around 15 min per dyad.

### fNIRS Acquisition and Preprocessing

**FIGURE 1:**
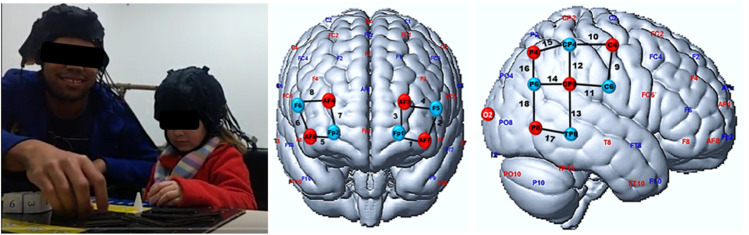
fNIRS montage. (Left) Participants during an experiment with fNIRS sensors attached (Middle and Right). fNIRS cortical measurement locations visualized on the brain surface, on prefrontal and temporal-parietal cortices. Red and blue circles represent light sources and detectors, respectively. EEG 10—10 international system positions are also depicted. The black lines and numbers are the fNIRS channels. The same montage was used for both student and teacher heads.

We used a NIRScout (NIRx Medical Technologies, New York, NY, United States) sampling rate of 7.81 Hz device, with 16 sources and 16 detectors to simultaneously collect the teachers’ and students’ hemodynamics brain data. For each participant, optodes were positioned in the PFC (channels from 1 to 8) and the TPJ (channels from 9 to 18), Figure 1. The first was chosen because it is involved with executive functions related to counting and simple mathematical operations (Artemenko et al., 2018). The second is related to social features such as empathy and memorization (Brockington et al., 2018). The data was collected using NIRSTAR acquisition software. We preprocessed the fNIRS signals to reduce the effects of artifacts. First, we made a visual inspection to detect signals irregularities that could be related to artifacts or data collection problems. Data with irregularities such as missing channels and saturated values were discarded. Second, we applied a bandpass filter (0.01 Hz < freq. < 0.2 Hz) on the raw data to remove low-frequency systemic artifacts and cardiac and respiratory cycles. We then calculated the HBO_2_ variations by using the modified Beer-Lambert law with the whole time series as a baseline and differential path lengths (DPF) 7.25 and 6.38 for the wavelengths of 760 and 850 nm, respectively. Calculations were performed with a home-made MATLAB script from our research group.

### The Predictive Models

We used two regression models to predict the signals of students using the teachers’ signals as predictors. The first is the SVR, an approach used to create predictive models for continuous data. One of SVR’s advantages is the power to treat high dimensionality and multicollinearity data, providing greater prediction of unseen data (Awad and Khanna, 2015). The second is the traditional OLS, a more conservative approach that requires assumptions such as homoscedasticity and the absence of the residuals’ autocorrelation. These assumptions may not always be satisfied with fNIRS signals (Huppert, 2016).

Several studies of ML and fNIRS have demonstrated that model’s accuracy is higher when using HBO_2_ signals to classification and prediction models (Bogler et al., 2014; Song et al., 2016; Liu and Ayaz, 2018; Rojas et al., 2019). Therefore, the predictive models were performed over the HBO_2_ signals of students *S*_*i*_ and teachers *T*_*i*_,with *i* = 1,2,3…0.18 (number of fNIRS channels). We considered the whole task in the analysis, which is approximately 7,000 time-points (∼15 min × 7.81). We trained the models with the first 50% of the data $\left\{S_{i}^{t\,\text{r}},T_{i}^{t\,\text{r}}\right\}$, and the other 50% $\left\{S_{i}^{t\,\text{s}},T_{i}^{t\,\text{s}}\right\}\,$ was used for prediction (i.e., testing the models’ performance). For each pair of student-teacher, the SVR (with linear kernel) and OLS multivariate models were built with the student’s data from each channel *j* = 1,2,3…18 being the response variable, and the signals of the 18 teacher’s channels the predictors (Equation 1). It gave us one model for each fNIRS channel, resulting 18 models per student-teacher pair with prediction performed *via* SVR, and 18 models predicting *via* OLS. We applied them to the teachers’ test $T_{i}^{t\,\text{s}}$ data to predict the students’ signals $S_{i}^{p\,r}$ (Equation 2).

(1) $$S_{j}^{t\,r}=\sum_{i=1}^{18}\left(w_{i}*T_{i}^{t\,\text{r}}\right)+b$$

(2) $$S_{j}^{P\,r}=\sum_{i=1}^{18}\left(w_{i}*T_{i}^{t\,\text{s}}\right)+b$$

**FIGURE 2:**
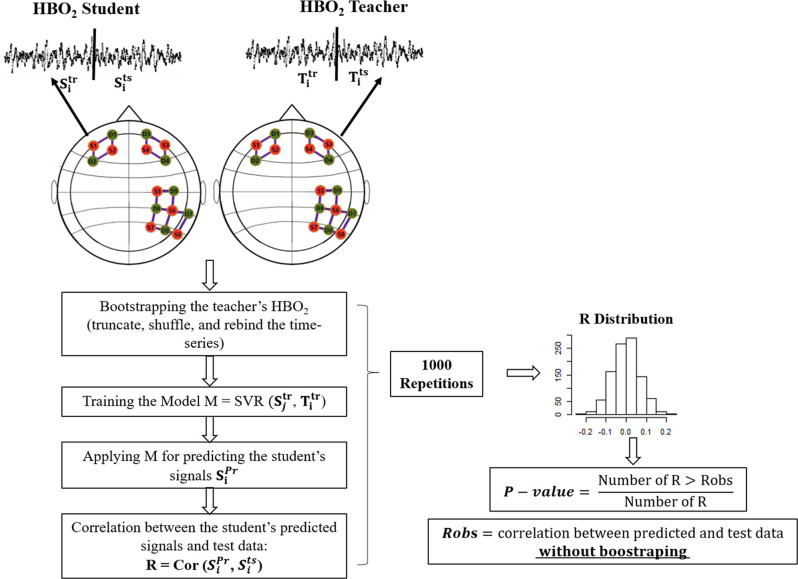
Scheme of the bootstrapping approach. The two circles depict the student and teacher’s head. Green and orange circles represent the fNIRS sources and detectors. Full lines represent channels. For simplicity, we only include the SVR in this picture (Training the Model). However, the procedure with OLS is analogous.

As the accuracy metric, we computed the Spearman correlation coefficient (which is robust against outliers) between the predicted $S_{i}^{p\,r}\,$ and the test $S_{i}^{t\,s}$ signals of the students, for each fNIRS channel. We tested the statistical significance of the correlation *via* a null distribution built by using a bootstrap approach (see Figure 2). We first found the lag in which the autocorrelation of the teacher’s HBO_2_ time-series were close to zero. The lag varied for each teacher-student dyad being (35, 37, 30, 51, and 83 points), for the respective dyads (1,2,3,4,5). We used this value to truncate the teacher’s time series in blocks, following the rule $n\,u\,m\,b\,e\,r\,o\,f\,b\,l\,o\,c\,k\,s=\frac{t\,i\,m\,e\,s\,e\,r\,i\,e\,s\,l\,e\,n\,g\,t\,h}{l\,a\,g}$. The blocks were shuffled and rebound. This procedure minimized the temporal dependency between the teachers’ and students’ signals. The training, predicting, and testing modeling were repeated 1,000 times with the teacher’s resampled HBO_2_ signals as the predictors; and a null distribution of the correlations coefficients was built. The *p*-value was calculated as the ratio between the values computed with bootstrapped data higher than the calculated with original signals, and the total number of coefficients computed with bootstrapped data. The SVR computations were performed using the package e1071 of the R software (Meyer et al., 2019). A scheme describing the procedure is depicted in Figure 2.

## Results

**FIGURE 3:**
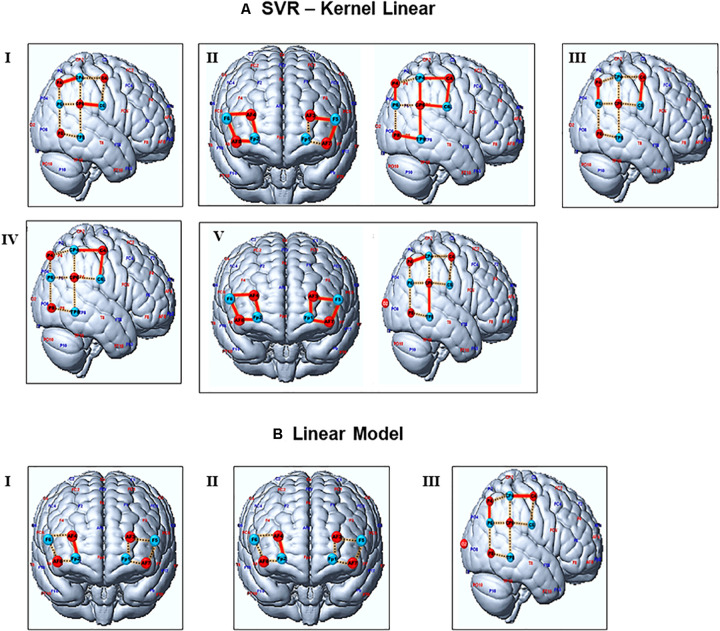
Representation of channels with significant signal’s predictions. **(A,B)** depict the channels predicted by the SVR and OLS models, respectively. Red lines represent channels with a statistically significant correlation between test and predicted data (without FDR corrections). Channels with significant outcomes after FDR corrections are presented in bold letters at Table 1. Dashed yellow represents the general channels. Blue and red circles are the source and detectors. Numbers from I to V represent the student-teacher dyads.

The student’s signals predicted with the SVR model $S_{j}^{P\,r}$ were statistically significantly correlated with the measured test data recordings $S_{i}^{t\,s}$ for all five teacher-student dyads, for a significance level of 0.01 (Figure 3A). A few channels lost their significant results after a false discovery rate (FDR) correction. Considering the uncorrected values, we found correlations in the signals from different channels located in the TPJ. All dyads had at least two signals from channels of this region correlated with predicted signals. For instance, signals from channels 11 and 15 of dyad I, channels 9 and 16 of dyad III, channels 9 and 10 of dyads IV, and channels 13 and 15 of dyad V showed these results. Dyad II had signals from almost all channels (9,10,11,12,13,16, and 17) of the TPJ associated with the predicted data; the only exception was the channels 14,15 and 18. The SVR predictions of signals from the prefrontal cortex were significantly correlated to the test data of dyad II and V. These results were verified in almost all channels of both dyads, except for channels 1 and 3 of dyad II; see Table 1. However, for the correlation corrected values (FDR, significance level = 0.01), dyads II and V kept the significant results, while the few outcomes related to dyads I, III, and IV lost the statistical significance.

**TABLE 1:** Correlation between SVR and OLS predictions and test data.

Channel	Dyad I	Dyad II	Dyad III	Dyad IV	Dyad V
1					0.25**
2		0.28**			0.23*
3					0.30**
4		0.26**			0.18*
5		0.31**			0.36**
6		0.18*			0.22*
7	0.12* (OLS)	0.17* 0.14* (OLS)			0.33**
8		0.24**			0.19*
9		0.29**	0.15*	0.17*	
10		0.31**	0.23* (OLS)	0.22*	
11	0.18*	0.30**			
12		0.23**			
13		0.23**			
15	0.15*				0.21*
16		0.28**	0.16* 0.20* (OLS)		
17		0.30**			

Spearman coefficients of correlations between the signals predicted and test data. The OLS predictions have the abbreviation (OLS) next to the value of correlation. Only statistically significant results are presented; ** *p*-value ≤ 0.001; *p-value ≤ 0.01. Bold letters represent channels with significant outcomes after FDR corrections.

On the other hand, only a few predictions performed with OLS showed significant results, for a significance level of 0.01 (Figure 3B). Predicted signals of the prefrontal cortex (channel 7) from pairs I and II were statically significantly correlated with the real data, while pair III showed this outcome in channels 10 and 16 from the TPJ. The OLS predictions showed significant associations with real data only in three out of five pairs of subjects, while SVR predicted associations for all pairs of however, the statistical significance of these results does not survive after the FDR correction.

Some channels showed negative correlations between SVR/OLS predictions and test data. However, these values are not statistically significant. The Spearman coefficients of correlations between OLS and SVR predictions and test data, with their corrected and uncorrected *p*-values, are shown in the Supplementary Material. Tables 2 and 3, respectively.

**TABLE 2:** Spearman Correlation between OLS predictions and Test data.

Ch	Dyad 1			Dyad 2			Dyad 3			Dyad 4			Dyad 5		
	S	P	FDR	S	P	FDR	S	P	FDR	S	P	FDR	S	P	FDR
1	–0.039	0.78	1.00	0.056	0.66	0.89	0.029	0.34	0.50	0.002	0.53	0.92	0.024	0.30	0.94
2	–0.043	0.74	1.00	0.041	0.32	0.89	0.156	0.04	0.13	0.067	0.25	0.92	–0.133	0.80	0.94
3	–0.036	0.60	1.00	–0.026	0.79	0.89	0.053	0.06	0.17	0.101	0.21	0.92	–0.064	0.47	0.94
4	–0.075	0.93	1.00	–0.026	0.70	0.89	0.111	0.23	0.46	0.069	0.31	0.92	–0.110	0.62	0.94
5	–0.008	0.33	1.00	0.081	0.07	0.62	0.157	0.03	0.13	–0.074	0.37	0.92	–0.147	0.92	0.94
6	–0.062	0.86	1.00	–0.038	0.50	0.89	–0.043	0.84	0.89	0.007	0.46	0.92	–0.152	0.90	0.94
7	0.117	0.01	0.13	0.140	<0.01	0.05	0.030	0.36	0.50	–0.037	0.33	0.92	–0.173	0.90	0.94
8	0.031	0.30	1.00	–0.058	0.51	0.89	0.016	0.75	0.84	–0.165	0.97	0.97	–0.124	0.85	0.94
9	–0.110	0.90	1.00	–0.077	0.76	0.89	0.039	0.43	0.52	–0.070	0.75	0.97	–0.193	0.91	0.94
10	0.005	0.30	1.00	–0.026	0.44	0.89	0.226	<0.01	0.04	0.156	0.10	0.92	–0.022	0.36	0.94
11	–0.125	0.97	1.00	0.021	0.49	0.89	0.149	0.10	0.24	–0.132	0.92	0.97	–0.130	0.94	0.94
12	–0.193	0.95	1.00	–0.048	0.49	0.89	0.179	0.03	0.13	–0.104	0.96	0.97	–0.103	0.82	0.94
13	–0.030	0.69	1.00	–0.068	0.77	0.89	–0.229	1.00	1.00	–0.080	0.81	0.97	–0.061	0.60	0.94
14	–0.086	0.80	1.00	0.011	0.56	0.89	–0.013	0.37	0.50	–0.052	0.56	0.92	–0.036	0.36	0.94
15	–0.096	0.92	1.00	–0.137	1.00	1.00	0.059	0.26	0.47	–0.097	0.84	0.97	–0.062	0.67	0.94
16	–0.217	1.00	1.00	–0.015	0.60	0.89	0.200	<0.01	0.04	–0.054	0.69	0.97	–0.080	0.87	0.94
17	0.029	0.31	1.00	–0.014	0.49	0.89	0.070	0.39	0.50	0.051	0.26	0.92	–0.088	0.61	0.94
18	–0.012	0.42	1.00	–0.110	0.89	0.94	0.066	0.11	0.24	–0.030	0.48	0.92	–0.106	0.69	0.94

*Abbreviations in the table stand for: Ch, Number of the fNIRS Channel; S, Spearman Correlation between the predicted (S_*i*_^*pr*^) and the test (S_*i*_^*ts*^) signals; P, *P*-value of the Spearman correlation; FDR, P-value corrected by the False Discovery Rate (FDR); Underlined numbers, *P*-value ≤ 0.01.*

**TABLE 3:** Correlation between SVR predictions and Test data.

Ch	Dyad 1			Dyad 2			Dyad 3			Dyad 4			Dyad 5		
	S	P	FDR	S	P	FDR	S	P	FDR	S	P	FDR	S	P	FDR
1	–0.010	0.57	0.72	0.083	0.14	0.17	0.094	0.06	0.18	0.139	0.04	0.162	0.246	<0.01	0.00
2	–0.090	0.91	0.91	0.283	<0.01	0.00	–0.004	0.51	0.66	0.072	0.20	0.321	0.228	<0.01	0.01
3	–0.012	0.60	0.72	–0.007	0.55	0.58	0.057	0.22	0.42	0.088	0.13	0.299	0.294	<0.01	0.00
4	0.031	0.30	0.68	0.262	<0.01	0.00	–0.007	0.52	0.66	0.109	0.10	0.255	0.178	0.01	0.02
5	0.005	0.50	0.70	0.311	<0.01	0.00	0.068	0.16	0.35	–0.087	0.89	0.94	0.360	<0.01	0.00
6	–0.067	0.86	0.91	0.185	<0.01	0.00	0.092	0.08	0.20	0.139	0.05	0.162	0.216	<0.01	0.01
7	0.013	0.46	0.70	0.174	0.01	0.01	–0.055	0.80	0.84	0.078	0.17	0.321	0.331	<0.01	0.00
8	0.022	0.39	0.70	0.236	<0.01	0.00	0.031	0.32	0.53	0.057	0.21	0.321	0.195	<0.01	0.01
9	0.120	0.04	0.26	0.293	<0.01	0.00	0.154	0.01	0.11	0.171	0.01	0.108	0.061	0.21	0.27
10	0.016	0.42	0.70	0.309	<0.01	0.00	–0.039	0.73	0.82	0.219	<0.01	0.036	0.060	0.23	0.27
11	0.176	0.01	0.06	0.300	<0.01	0.00	–0.234	1.00	1.00	–0.040	0.70	0.791	–0.276	1.00	1.00
12	0.044	0.22	0.67	0.228	<0.01	0.00	0.113	0.04	0.18	0.056	0.20	0.321	0.127	0.04	0.07
13	0.080	0.12	0.43	0.232	<0.01	0.00	0.113	0.06	0.18	–0.023	0.62	0.745	0.099	0.10	0.15
14	0.000	0.50	0.70	0.067	0.15	0.18	0.132	0.03	0.17	–0.152	0.98	0.979	0.009	0.46	0.52
15	0.152	0.01	0.06	–0.024	0.64	0.64	0.044	0.23	0.42	0.034	0.35	0.485	0.215	<0.01	0.01
16	0.090	0.09	0.42	0.276	<0.01	0.00	0.161	<0.01	0.05	0.004	0.50	0.638	–0.025	0.64	0.68
17	0.036	0.30	0.68	0.301	<0.01	0.00	–0.011	0.59	0.71	0.109	0.09	0.255	0.064	0.21	0.27
18	–0.033	0.70	0.79	0.032	0.30	0.34	0.007	0.45	0.66	0.138	0.03	0.162	0.159	0.02	0.04

*Abbreviations in the table stand for: Ch, Number of the fNIRS Channel; S, Spearman Correlation between the predicted (S_*i*_^*pr*^) and the test (S_*i*_^*ts*^) signals; P, *P*-value of the Spearman correlation; FDR, P-value corrected by the False Discovery Rate (FDR); Underlined numbers, *P*-value ≤ 0.01.*

## Discussion

This study aimed to test whether the teachers’ signals can predict a students’ brain hemodynamic. We applied the machine learning algorithm SVR and compared the results with predictions performed *via* the traditional OLS. The SVR yielded significant results for all dyads, while OLS presented statistically significant correlations with the test data of only two. The results with SVR and the OLS differed in the number of dyads and fNIRS channels. SVR predicted a total of 29/90 signals across the five pairs of individuals (18 channels x 5 dyads = 90 signals), while OLS predicted only 4/90. When considering multiple corrections, these numbers go to 23/90 for the SVR, and no significant results for OLS.

The fact that SVR predicted more statistically significant results than the OLS might be explained by the conceptualization of its estimator. It follows the principle of maximal margin. It does not care about the prediction as long as the error is less than, which is the highest deviation of the prediction function *f*(*x*) from the target value *yi*. These features, combined with the fact that the cost parameter can penalize the regression, provide the SVR power to avoid over-fitting and give more generalization to the test data (Smola and Schölkopf, 2004; Awad and Khanna, 2015). These finds are supported by other studies that used SVR to predict hemodynamic brain signals. For instance, Liu et al. (2015) argued that SVR is more suitable than OLS to predict human deep-brain regions’ activity using fNIRS since SVR defines the weights to reflect the contributions of the features better than the OLS. Zhang et al. (2014) compared the SVR and OLS application to synthetic data to predict voxel-based lesion-symptom mapping (VLSM). They verified that SVR presented higher sensitivity and specificity for detecting the lesion-behavior relationship than the OLS.

This proof-of-concept study is focused on developing and testing a new methodological approach and not designed to investigate the specific brain areas involved in the teaching-learning process. However, it is relevant to note that all dyads showed a relationship between training and testing data of the TPJ, a brain area known to be involved in social cognition and processes underlying empathy and social interactions (Zheng et al., 2018). For instance, Zheng et al. (2018) found that interpersonal neural synchronization (INS) between the student’s and teacher’s TPJ varied with the teaching strategy; an increase of INS between the right TPJ of the teacher and anterior superior temporal cortex of the student was associated to better teaching outcome. The fMRI study about predictions of human decisions in a poker game showed that signals from the TPJ provided unique information about the upcoming decision (Carter et al., 2013). Based on that, our finds give evidence to confirm that this region plays a fundamental role in the cognition process underlying student-teacher interaction.

On the other hand, only two out of the five pairs presented statistically significant correlations between training and test data from the pre-frontal cortex. This area is related to the cognitive process related to learning and has been evaluated with fNIRS in diverse tasks before (Wood and Grafman, 2003; Ayaz et al., 2012; Singh et al., 2018; Nozawa et al., 2019). Additionally, when performing the task, the dyads recruits several executive functions such as attention and inhibitory control during the verbal communication. Those functions are related to the PFC activity (Gvirts and Perlmutter, 2020; Kelsen et al., 2020). Furthermore, considering that our task consists of a teaching-learning process of adding two numbers less or equal to six, the discrepant results across the dyads might be explained by the differences in the cognitive workload of each child performing the task. It may require different engagement levels with the teachers for learning how to add the numbers leading to the different results found here (Sun et al., 2020; Zhang et al., 2020).

Some limitations must be considered in this study. The sample size is small so that more studies with a higher number of participants are necessary regarding the generalization of the results. We did not have 3D-digitizers to map the optodes locations on the participants’ heads. The use of 3D-digitizers in Pinti et al. (2019) follow-up study could add more information for comparing homologous brain areas and homogeneity of the regions across subjects. Although short-channels data contribute to reducing physiological noise, we did not perform this measurement due to our fNIRS device limitations. Nonetheless, we tried to reduce those effects by applying filters to our data (Yücel et al., 2021). We adopted the conservative band-pass approach to filter the fNIRS data and avoid excessive modifications in the signals, which could mask relevant aspects during the prediction procedures. This choice was made because different filtering methods might interfere with the outcomes (Huppert, 2016; Pinti et al., 2019). While we applied band-pass filter to reduce the physiological noise, the fNIRS signal can be still confounded by motion artifacts. Therefore, other filters might be useful according to the features of the data (Brigadoi et al., 2014). Additionally, given the limited number of sensors, we could only investigate cortical regions within the prefrontal and right TPJ regions. Nevertheless, other areas may also play a role in the teacher-student interaction, and future studies may explore other cortical areas with high-density sensors. Also, fNIRS provides information about cortical areas, restricting the investigation of subcortical regions that may also be relevant to the teacher-student interaction (Kostorz et al., 2020). Additional physiological signals have been shown not to contribute to the mental state decoding (Liu et al., 2017). However, such signals (e.g., heart rate, heart rate variability, and skin conductance) could bring relevant information about the participant’s arousal in this context and contribute to the prediction model.

Our proposed methodology demonstrated the possibility of using the teacher’s fNIRS signals to predict the student’s brain hemodynamic response. According to previous work, teaching outcomes are improved according to the teacher-student brain synchronization, and it is theoretically supported by the prediction-transmission hypothesis (Kline, 2015; Zheng et al., 2018). Preliminary results suggest that our proposed approach can be used to better understand the brain synchronization during the teacher-student interaction in which, speculatively, the teacher and student behaviors may be continuously updated according to their brain state predictions. Regardless, future research with a larger sample size and a broader number of fNIRS should continue to investigate which brain areas of the teacher are related to the students’ brain prediction. It can be achieved by considering the weights/contribution of each channel in teacher’s cohort in/to predicting student’s brain response. It will add more information about the neural mechanisms underlying the teaching-learning process and give experimental evidence for theoretical frameworks such as the prediction-transmission hypothesis.

## Data Availability Statement

The datasets presented in this article are not readily available because the ethics committee did not permit the sharing of data. Requests to access the datasets should be directed to JS, joao.sato@ufabc.edu.br.

## Ethics Statement

The studies involving human participants were reviewed and approved by Comite de Etica em Pesquisa (CEP)—Universidade Federal do ABC, SP-Brazil. Written informed consent to participate in this study was provided by the participants’ legal guardian/next of kin. Written informed consent was obtained from the individual(s), and minor(s)’ legal guardian/next of kin, for the publication of any potentially identifiable images or data included in this article.

## Author Contributions

CB collected, analyzed the data, and wrote the manuscript. GAB participated in the conception of the experiment and data collection. GB participated in the conception of the experiment and data collection. HA revised and contributed to improving the quality of the manuscript. JS conceived the experiment, supervised the data collection and analysis, revised the manuscript, and contributed to improving the quality of the manuscript. All authors contributed to the article and approved the submitted version.

## Conflict of Interest

The authors declare that the research was conducted in the absence of any commercial or financial relationships that could be construed as a potential conflict of interest.
