# Supplementary material for: A New Statistical Approach for fNIRS Hyperscanning to Predict Brain Activity of Preschoolers’ Using Teacher’s
Source: Front Hum Neurosci. 2021 May 7;15:622146. doi: 10.3389/fnhum.2021.622146 (PMC8137814; doi:10.3389/fnhum.2021.622146)
Supplement: Supplementary file 1 [file Data_Sheet_1.PDF]

Table 2: Spearman Correlation between OLS predictions and Test data

Ch	Dyad 1			Dyad 2			Dyad 3			Dyad 4			Dyad 5		
	**S**	**P**	**FDR**	**S**	**P**	**FDR**	**S**	**P**	**FDR**	**S**	**P**	**FDR**	**S**	**P**	**FDR**
1	-0.039	0.78	1.00	0.056	0.66	0.89	0.029	0.34	0.50	0.002	0.53	0.92	0.024	0.30	0.94
2	-0.043	0.74	1.00	0.041	0.32	0.89	0.156	0.04	0.13	0.067	0.25	0.92	-0.133	0.80	0.94
3	-0.036	0.60	1.00	-0.026	0.79	0.89	0.053	0.06	0.17	0.101	0.21	0.92	-0.064	0.47	0.94
4	-0.075	0.93	1.00	-0.026	0.70	0.89	0.111	0.23	0.46	0.069	0.31	0.92	-0.110	0.62	0.94
5	-0.008	0.33	1.00	0.081	0.07	0.62	0.157	0.03	0.13	-0.074	0.37	0.92	-0.147	0.92	0.94
6	-0.062	0.86	1.00	-0.038	0.50	0.89	-0.043	0.84	0.89	0.007	0.46	0.92	-0.152	0.90	0.94
7	0.117	0.01	0.13	0.140	<0.01	0.05	0.030	0.36	0.50	-0.037	0.33	0.92	-0.173	0.90	0.94
8	0.031	0.30	1.00	-0.058	0.51	0.89	0.016	0.75	0.84	-0.165	0.97	0.97	-0.124	0.85	0.94
9	-0.110	0.90	1.00	-0.077	0.76	0.89	0.039	0.43	0.52	-0.070	0.75	0.97	-0.193	0.91	0.94
10	0.005	0.30	1.00	-0.026	0.44	0.89	0.226	<0.01	0.04	0.156	0.10	0.92	-0.022	0.36	0.94
11	-0.125	0.97	1.00	0.021	0.49	0.89	0.149	0.10	0.24	-0.132	0.92	0.97	-0.130	0.94	0.94
12	-0.193	0.95	1.00	-0.048	0.49	0.89	0.179	0.03	0.13	-0.104	0.96	0.97	-0.103	0.82	0.94
13	-0.030	0.69	1.00	-0.068	0.77	0.89	-0.229	1.00	1.00	-0.080	0.81	0.97	-0.061	0.60	0.94
14	-0.086	0.80	1.00	0.011	0.56	0.89	-0.013	0.37	0.50	-0.052	0.56	0.92	-0.036	0.36	0.94
15	-0.096	0.92	1.00	-0.137	1.00	1.00	0.059	0.26	0.47	-0.097	0.84	0.97	-0.062	0.67	0.94
16	-0.217	1.00	1.00	-0.015	0.60	0.89	0.200	<0.01	0.04	-0.054	0.69	0.97	-0.080	0.87	0.94
17	0.029	0.31	1.00	-0.014	0.49	0.89	0.070	0.39	0.50	0.051	0.26	0.92	-0.088	0.61	0.94
18	-0.012	0.42	1.00	-0.110	0.89	0.94	0.066	0.11	0.24	-0.030	0.48	0.92	-0.106	0.69	0.94

Ch = Channel

S = Spearman Correlation between the predicted (S_i^{pr}) and the test (S_i^{ts}) signals

P = P-value of the Spearman correlation

Underlined numbers = P-value ≤0.01
