# Supplementary material for: A New Statistical Approach for fNIRS Hyperscanning to Predict Brain Activity of Preschoolers’ Using Teacher’s
Source: Front Hum Neurosci. 2021 May 7;15:622146. doi: 10.3389/fnhum.2021.622146 (PMC8137814; doi:10.3389/fnhum.2021.622146)
Supplement: Supplementary file 2 [file Data_Sheet_2.PDF]

Table 3: Correlation between SVR predictions and Test data

Ch	Dyad 1			Dyad 2			Dyad 3			Dyad 4			Dyad 5		
	S	P	FDR	S	P	FDR	S	P	FDR	S	P	FDR	S	P	FDR
1	-0.010	0.57	0.72	0.083	0.14	0.17	0.094	0.06	0.18	0.139	0.04	0.162	0.246	<0.01	0.00
2	-0.090	0.91	0.91	0.283	<0.01	0.00	-0.004	0.51	0.66	0.072	0.20	0.321	0.228	<0.01	0.01
3	-0.012	0.60	0.72	-0.007	0.55	0.58	0.057	0.22	0.42	0.088	0.13	0.299	0.294	<0.01	0.00
4	0.031	0.30	0.68	0.262	<0.01	0.00	-0.007	0.52	0.66	0.109	0.10	0.255	0.178	0.01	0.02
5	0.005	0.50	0.70	0.311	<0.01	0.00	0.068	0.16	0.35	-0.087	0.89	0.94	0.360	<0.01	0.00
6	-0.067	0.86	0.91	0.185	<0.01	0.00	0.092	0.08	0.20	0.139	0.05	0.162	0.216	<0.01	0.01
7	0.013	0.46	0.70	0.174	0.01	0.01	-0.055	0.80	0.84	0.078	0.17	0.321	0.331	<0.01	0.00
8	0.022	0.39	0.70	0.236	<0.01	0.00	0.031	0.32	0.53	0.057	0.21	0.321	0.195	<0.01	0.01
9	0.120	0.04	0.26	0.293	<0.01	0.00	0.154	0.01	0.11	0.171	0.01	0.108	0.061	0.21	0.27
10	0.016	0.42	0.70	0.309	<0.01	0.00	-0.039	0.73	0.82	0.219	<0.01	0.036	0.060	0.23	0.27
11	0.176	0.01	0.06	0.300	<0.01	0.00	-0.234	1.00	1.00	-0.040	0.70	0.791	-0.276	1.00	1.00
12	0.044	0.22	0.67	0.228	<0.01	0.00	0.113	0.04	0.18	0.056	0.20	0.321	0.127	0.04	0.07
13	0.080	0.12	0.43	0.232	<0.01	0.00	0.113	0.06	0.18	-0.023	0.62	0.745	0.099	0.10	0.15
14	0.000	0.50	0.70	0.067	0.15	0.18	0.132	0.03	0.17	-0.152	0.98	0.979	0.009	0.46	0.52
15	0.152	0.01	0.06	-0.024	0.64	0.64	0.044	0.23	0.42	0.034	0.35	0.485	0.215	<0.01	0.01
16	0.090	0.09	0.42	0.276	<0.01	0.00	0.161	<0.01	0.05	0.004	0.50	0.638	-0.025	0.64	0.68
17	0.036	0.30	0.68	0.301	<0.01	0.00	-0.011	0.59	0.71	0.109	0.09	0.255	0.064	0.21	0.27
18	-0.033	0.70	0.79	0.032	0.30	0.34	0.007	0.45	0.66	0.138	0.03	0.162	0.159	0.02	0.04

Ch = Channel

S = Spearman Correlation between the predicted (S_i^{pr}) and the test (S_i^{ts}) signals

P = P-value of the Spearman correlation

FDR = P-value corrected by the False Discovery Rate (FDR)

Underlined numbers = P-value ≤0.01
